# Cell culture-derived HCV cannot infect synovial fibroblasts

**DOI:** 10.1038/srep18043

**Published:** 2015-12-08

**Authors:** Abd-Elshafy D. Nadeem, Pietschmann Thomas, Müller-Ladner Ulf, Neumann Elena, A Anggakusuma, Bahgat M. Mohamed, Pessler Frank, Behrendt Patrick

**Affiliations:** 1 TWINCORE, Center for Experimental and Clinical Infection Research, Institute for Experimental Infection Research, Hannover, Germany; 2 Water Pollution Research Department, Environmental Sciences Research Division, National research center, Cairo, Egypt; 3 TWINCORE, Center for Experimental and Clinical Infection Research, Institute of Experimental Virology, Hannover, Germany; 4 German Centre for Infection Research, Hannover, Germany; 5 Department of Internal Medicine and Rheumatology, Justus-Liebig University of Giessen, Rheumatology and Clinical Immunology, Kerckhoff Klinik, GmbH, Bad Nauheim, Germany; 6 Therapeutic Chemistry Department, Pharmaceutical Industries Research Division, National research center, Cairo, Egypt; 7 Immunology and lnfectious Diseases Laboratory, Therapeutic Chemistry Department, the National Research Centre, Cairo, Egypt; 8 Helmholtz Centre for Infection Research (HZI), Braunschweig, Germany; 9 Department for Gastroenterology, Hepatology and Endocrinology, Medical School Hannover, Hannover, Germany

## Abstract

Worldwide 170 million individuals are infected with hepatitis C virus (HCV), up to 45 million of whom are affected by arthropathy. It is unclear whether this is due to viral infection of synovial cells or immune-mediated mechanisms. We tested the capacity of primary synovial fibroblasts to support HCV propagation. Out of the four critical HCV receptors, only CD81 was expressed to any significant extent in OASF and RASF. Consistent with this, pseudotyped HCV particles were unable to infect these cells. Permissiveness for HCV replication was investigated by transfecting cells with a subgenomic replicon of HCV encoding a luciferase reporter. OASF and RASF did not support replication of HCV, possibly due to low expression levels of miR-122. In conclusion, primary human synovial fibroblasts are unable to support propagation of HCV *in vitro*. HCV-related arthropathy is unlikely due to direct infection of these cells.

It is estimated that more than 80% of individuals infected with hepatitis C virus (HCV) remain persistently infected, and approximately 20–40% of them develop cirrhosis with a possible progression to hepatocellular carcinoma^1^,^2^. Although most chronically infected individuals are asymptomatic, an appreciable number will experience symptoms due to liver disease and/or extra-hepatic manifestations^3^. Extrahepatic manifestations have been reported in 40–74% of HCV infected individuals, and are mainly believed to be immune-mediated. Fatigue is the most common symptom, but involvement of skin, eyes, kidneys, the central and peripheral nervous systems, the immune system, and the musculoskeletal system (including synovial joints) is reported^4^,^5^,^6^.

HCV infection-associated arthritis (HAA) often presents as a symmetrical inflammatory arthritis involving small joints and may thus clinically resemble rheumatoid arthritis (RA). In addition, rheumatoid factor (RF) is detected in 50–85% of patients with HAA, which often makes it difficult to differentiate between RA and HAA^7^,^8^. However, HAA is usually a non-deforming, non-erosive arthritis^9^. Its frequency has been studied in previous reports. A prospective study performed in France showed that 23% of HCV patients developed arthralgia and according to a study from Egypt the prevalence of all rheumatologic manifestations among HCV patients was 16.4% and frank arthritis was found in 0.7%^7^,^10^. When synovial biopsies from patients with HAA, osteoarthritis (OA), RA, and healthy controls were compared, it appeared that HAA may have distinct histopathological, and thus pathogenetic, features^11^. However, the pathophysiological mechanisms of HAA remain unsolved. In particular, it remains unclear whether HAA is due to direct infection of synovial cells by HCV or whether it results from immune-mediated long-distance effects. On one hand, HCV has been detected in extrahepatic cells such as peripheral blood mononuclear cells, dendritic cells, and even in the central nervous system^12^,^13^,^14^. Indeed, persistence of the virus in extra-hepatic sites is considered a major determinant of reinfection of hepatocytes in patients after liver transplantation for HCV-associated liver cirrhosis^15^. It is therefore conceivable that the synovial membrane may serve as an additional extrahepatic site to support HCV infection, leading to HAA.

On the other hand, attempts to detect HCV in the synovial fluid of a small number of patients suffering from both HCV infection and arthritis have mostly yielded negative results^16^. However, due to the non-erosive nature of HAA, surgically removed joint tissue usually does not become available, and synovial biopsies are usually not obtained from patients being evaluated for HAA. A systematic search for HCV in synovial tissue from patients with HAA has therefore not been possible and is unlikely to take place in the foreseeable future. As a first non-invasive step toward testing the hypothesis that HAA may be due to infection of resident synovial cells, we have therefore studied the capacity of OA synovial fibroblasts from patients with RA (RASF) and OA (OASF) to support HCV entry and replication.

## Material and Methods

### Cell Culture

Primary OASF and RASF were established as described and used between passages four and six^17^. OASF, RASF, Huh-7.5 and Hep-56.1D cells were cultured in Dulbecco’s modified Eagle’s medium (DMEM; Invitrogen, Karlsruhe, Germany) supplemented with 20% fetal bovine serum (FBS; Invitrogen), 1% HEPES (Invitrogen), 1% nonessential amino acids (Invitrogen), 100 μg/mL of streptomycin (Invitrogen), and 100 IU/mL of penicillin.

### Reporter HCV replicons and plasmids

Luc-NS3-5B is a bicistronic, subgenomic JFH1 replicon of the genotype 2a strain JFH1 which encodes a firefly luciferase gene and is generated by T7 polymerase-based *in vitro* transcription of the plasmid pFKi389Luc-EI/NS3-3′_JFH1_dg^18^. Luc-NS3-NS5BΔGDD is a derivative of this replicon which, in the NS5B gene, contains an in-frame deletion of 10 amino acids encompassing the conserved GDD motif, leading to loss of polymerase activity. The murine leukemia virus (MLV)-based retroviral vector pRV-F-Luc-IZ is a derivative of pczCFG5-IZ^19^. Briefly, this vector carries a chimeric 5′LTR in which the MLV-U3 region was replaced by a cytomegalovirus (CMV) immediate early promoter. The firefly luciferase transgene is inserted into the env locus of the original MLV genome and is followed by the internal ribosome entry site of encephalomyocarditis virus (EMCV) and the ShBle resistance gene conferring resistance to zeocin (Invitrogen, Karlsruhe, Germany).

### RNA quantification of the HCV receptors (CD81, SR-BI, claudin-1, and occludin) and miR-122

RNA was extracted from OASF, RASF and Huh-7.5 cells using the Nucleo Spin RNAII Kit (Macherey-Nagel; Düren, Germany). Equal RNA concentrations were then subjected to relative quantification of the transcripts encoding the HCV receptors using a Light Cycler 480 (Roche; Mannheim, Germany). Quantitative reverse transcriptase polymerase chain reaction (RT-PCR) employing mRNA-specific hydrolysis probes and appropriate primer sets was used to measure expression levels of the HCV-receptors CD81, scavenger receptor class B type I (SR-BI), claudin-1 (CLDN1) and occluding (OCLN). GAPDH was used as reference gene as described previously^20^. Additionally, we determined miR-122 expression levels as described before^19^.

### Detection of CD81 by flow cytometry

OASF, RASF, Huh-7.5 and Hep56 cells were scraped from confluent culture flasks, suspended in PBS and stained with CD81-specific antibodies (clone JS-81; Becton Dickinson, Heidelberg, Germany; 1:200) for 30 min at 4 ^o^C. Cells were washed and then incubated with anti-mouse Alexa 488 (Invitrogen; 1:200) for 30 min at 4 ^o^C. Cells were washed, fixed with 3% paraformaldehyde for 10 min, and analyzed immediately for surface expression of CD81 using a fluorescence-activated cell sorter (FACS Calibur, Becton-Dickinson). Data were analyzed using the FloJo software (Tree Star; Inc. Oregon Corporation, Ashland, OR).

### Detection of CLDN1, OCLN and SR-BI by Western blot analysis

Cells were washed once with PBS and lysed in sample buffer (400 mM Tris, pH 8.8, 10 mM EDTA, 0.2% bromophenol blue, 20% sucrose, 3% sodium dodecyl sulphate [SDS], 2% β-mercaptoethanol). Proteins were resolved by electrophoresis, transferred to a polyvinylidene difluoride membrane and then visualized using primary antibodies specific for CDLN1 (mouse anti-human CLDN1, clone 2H10D10, Zymed; Invitrogen), SR-BI (rabbit anti-human SR-BI, clone NB400-104, Novus Biologicals Littleton, CA) or OCLN (rabbit anti-human OCLN, clone OC-3F10, Zymed; Invitrogen). For detection of specific binding, appropriate HRP-conjugated secondary-antibodies were used (Sigma-Aldrich GmbH) and the ECL Plus peroxidase detection system was added for the development of peroxidase reaction (GE Healthcare Europe, Freiburg, Germany). For detection of β-actin, separate gels ran under the same experimental conditions. Thereafter, we used these blots to detect actin levels by addition of a HRP-coupled mouse anti-human β-actin antibody (Sigma-Aldrich GmbH). As a marker we used PageRuler PlusPrestained Prtoein Ladder (Thermo Scientific).

### Infection assay with retroviral pseudo-particles

We generated murine leukemia virus (MLV)-based pseudo-typed particles either harboring the envelope glycoproteins of vesicular stomatitis virus (VSV-G) or the glycoproteins of HCV (E1-E2 of clone J6CF, genotype 2a) as described previously^21^. An empty vector (pcDNA) served as negative control.

For infection assays, we seeded Huh-7.5 cells (1 × 10^5^ cells/ml), OASF or RASF (3.5 × 10^5^ cells/ml) in 12-well plates. Pseudo-particles were added after 24 h, and cells were lysed 48 h post infection using Passive Lysis Buffer (Promega). Infection was assessed by measuring luciferase activity as described later.

### *In vitro* transcription of plasmids encoding Luc-NS3-NS5B and Luc-NS3-NS5B∆GDD

Plasmid DNA purification, *in vitro* transcription, RNA purification, and testing RNA integrity were carried out as reported previously^22^.

### Cell transfection

OASF and RASF cells were seeded at a density of 1.3 × 10^5^ cells/ml, whereas Huh-7.5 cells were seeded at 1 × 10^5^ cells/ml in 12-well plates. After 24 h, cells were transfected with 1 μg RNA of the Luc-Jc1 reporter virus, Luc-Jc1ΔGDD or lacZ using Lipofectamine2000 reagent (Invitrogen) according to the manufacturer’s protocol. Medium was changed after 4 h. HCV RNA replication was quantified by measuring luciferase activity and detected by immunofluorescence staining of infected cells using FITC labeled anti-NS5A antibodies at 4 and 72 h post transfection.

### Luciferase assay

Transfected cells were washed twice with PBS and lysed in 300 μl per well luciferase lysis buffer (1% Triton X-100, 25 mM glycylglycine, 15 mM MgSO_4_, 4 mM EGTA and 1 mM DTT, pH 7.8). Cell lysates were stored at −20 ^o^C. A volume of 360 μl of assay buffer (0.5 M glycine, 0.1 M KPO_4,_ 1 M MgSO_4_, 0.2 M EGTA, 1 mM DTT, 2 mM ATP, pH 7.8) was added to each well before measuring. Firefly luciferase activity was measured with a luminometer (Lumat LB9507; Berthold, Freiburg, Germany).

### Indirect immunofluorescence

OASF, RASF and Huh-7.5 cells were seeded onto glass coverslips in 24-well plates at densities of 1.3 × 10^5^ cells/ml and 1 × 10^5^ cell/ml, respectively. Cells were fixed with 3% paraformaldehyde in PBS 72 h after transfection. Staining for NS5A was performed using 9E10 hybridoma supernatant (kindly provided by Dr. Charles Rice, Rockefeller University, New York) at a dilution of 1:2000 and anti-mouse Alexa flour 488 as secondary antibody^23^.

### X-Gal assay

OASF, RASF and Huh-7.5 cells, fixed with 3% paraformaldehyde in PBS 48 h post-transfection, were stained for 1–2 h with staining solution (1 mg/ml x-gal in DMF, 4 mM K_3_Fe(CN)_6_, 4 mM K_4_Fe(CN)_6_ and 2 mM MgCl_2_ × 6H_2_O in PBS), and blue clones were counted under an inverted microscope.

### Effect of OASF or RASF medium on infectivity of Huh-7.5

Seeded Huh-7.5 cells were transfected with 1 μg RNA of the Luc-Jc1 reporter virus, Luc-Jc1ΔGDD using Lipofectamine2000 reagent (Invitrogen) according to the manufacturer’s protocol. After 4 h, culture medium was replaced either with fresh medium or with supernatants from 5-day cultures of either OASF or RASF.

### Biological and experimental replicates

Protein and RNA extracts were prepared from seeded Huh-7.5, OASF and RASF cells in two individual flasks for each cell type and each extract was tested in duplicates in Western blotting and in triplicates in quantitative RT-PCR in two independent experiments to detect the expression of the receptors or the mRNAs encoding them. Thus, in Western blotting the total number of replicates was (n = 4) and in quantitative RT-PCR the total number of replicates was (n = 6). In each of the transfection experiments, Huh-7.5, OASF and RASF were seeded in triplicates and each experiment was repeated twice. Thus a total of 6 cell preparations (n = 6) from each transfected and control cell type were used in immunofluorescence detection. For flow cytometry, two independent detections of CD81 were done in triplicates on preparations from two independent cultures of the three cell types seeded in single flasks (n = 6). The pseudo-particle assay was performed twice, with two technical replicates each (n = 4).

## Results

### Host cell receptors for HCV are differentially expressed among the three cell types

**Figure 1:**
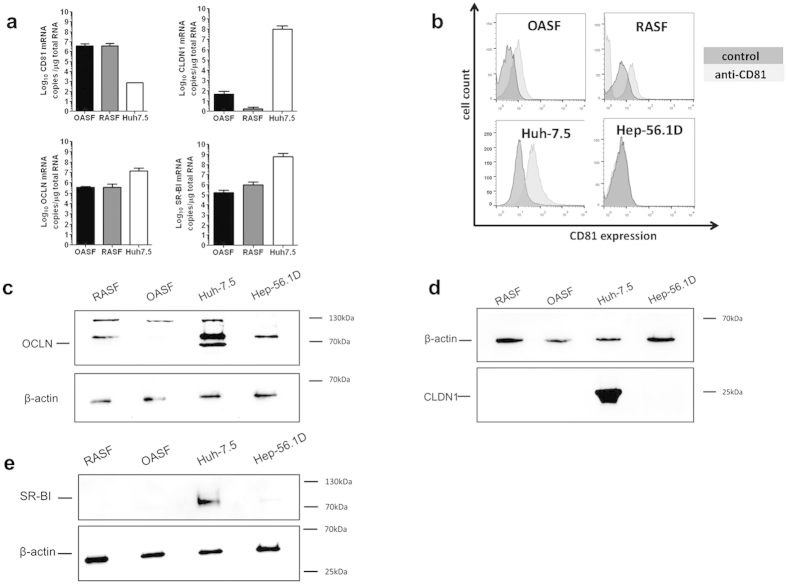
Expression of cellular HCV receptors at the mRNA and protein levels. (**a**) mRNA copy numbers of CD81, occluding (OCLN), claudin-1 (CLDN1) and SR-BI were quantified in OASF, RASF and Huh-7.5 cells, normalized to the reference gene GAPDH. (**b**) Detection of CD81 on Huh-7.5 cells, OASF, and RASF by FACS analysis. (**c–d**) Detection of OCLN, CLDN1 and SR-BI in OASF and RASF by immunoblot analysis. Huh-7.5 cells were used as positive control and Hep-56.1D as negative control. In both the real-time PCR and the FACS analysis experiments the number of biological replicates was 3 and the number of experimental replicates was 2 (total n = 6). In immunoblotting detection, the number of biological replicates was 2 and the number of experimental replicates was 2 (n = 4).

To explore permissiveness of OASF and RASF for HCV, we first explored the expression of the four critical HCV cell entry factors CD81, SR-BI, CLDN1 and OCLN^24^. CD81 mRNA was expressed in OASF and RASF at higher levels than in Huh-7.5 cells (Fig. 1a), whereas OCLN and SR-BI transcripts were expressed at lower levels in OASF and RASF than in Huh-7.5 cells. CLDN1 mRNA was detected at very low levels in OASF and was nearly undetectable in RASF (Fig. 1a). At the protein level, including Huh-7.5 cells as positive control and murine Hep-56.1D cells as negative control, FACS analysis showed that both OASF and RASF expressed CD81 (Fig. 1b). In contrast, CLDN1, SR-BI and OCLN could not be detected in OASF and RASF by immunoblotting (Fig. 1c–e).

### Synovial fibroblasts are resistant to HCV pseudo-particle infection

**Figure 2:**
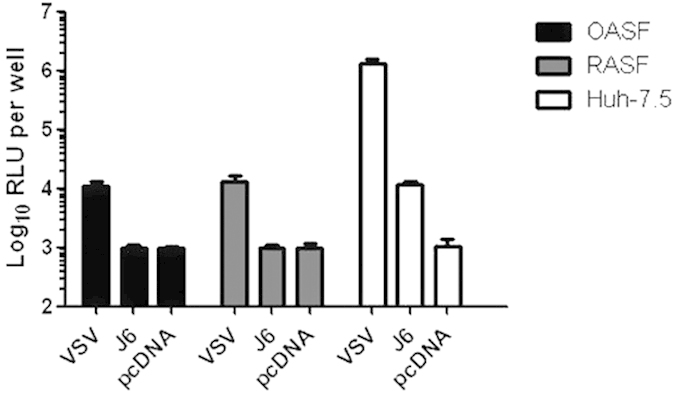
Infection assay of OASF, RASF and Huh-7.5 cells with retroviral pseudo-particles harboring the glycoproteins of vesicular stomatitis virus (VSV). Infection with pseudo-particles was carried out for 48 h. Firefly luciferase activity served as read out for infection. Transfection of pseudo-particle producing cells with the empty plasmid pcDNA served as a negative control and delineates the background level of this assay. The assay was conducted in two independent experiments with two technical replicates each.

To test whether OASF or RASF cells support HCV cell entry, we challenged them with retroviral pseudotypes carrying either VSV-G protein or J6CF-derived HCV genotype 2a glycoproteins. Retroviral particles without a viral envelope protein that were collected after transfection of pcDNA into 293T cells served as negative control for non-specific transduction of the luciferase-encoding retrovirus into the cells. MLV-based pseudo-particles harboring the glycoproteins of genotype 2a HCV infected Huh-7.5 cells but failed to infect OASF and RASF (Fig. 2). Pseudo-particles harboring the VSV envelope proteins efficiently infected Huh-7.5 and also OASF and RASF cells, although to a lesser extent (Fig. 2). Thus, OASF and RASF are permissive for infection by retroviral pseudotypes carrying VSV-G protein but not for pseudotypes bearing HCV E1-E2 proteins.

### OASF and RASF do not support replication of an HCV subgenomic replicon

**Figure 3:**
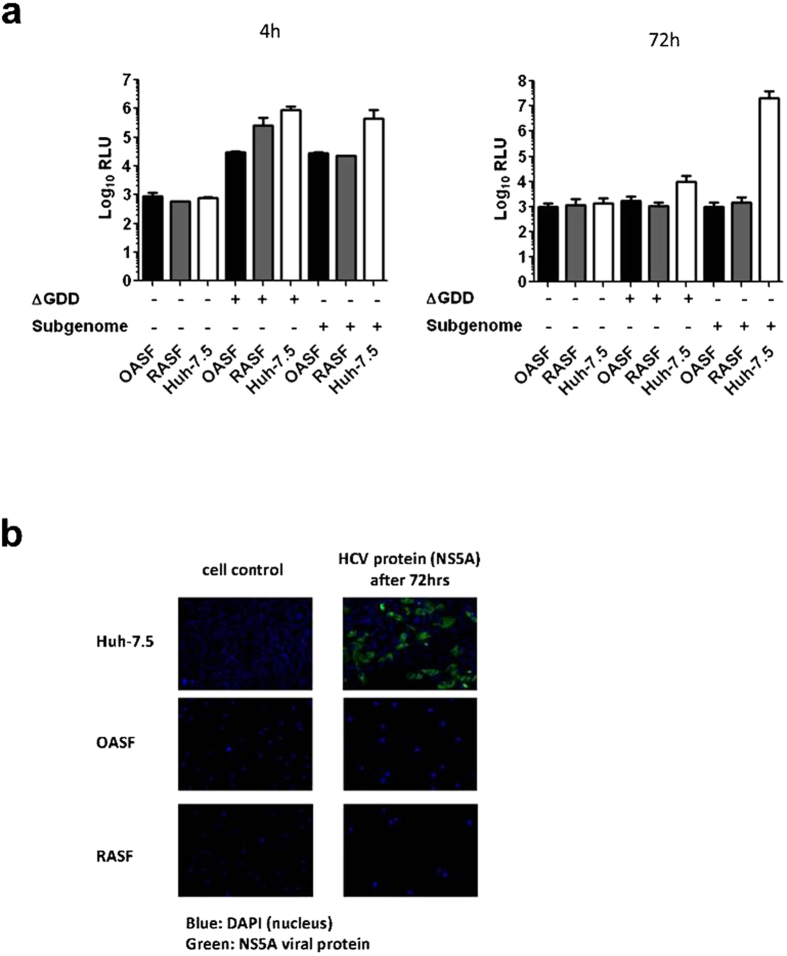
Monitoring replication of the HCV reporter subgenomic replicon in the synovial fibroblasts compared to Huh-7.5 cells. (**a**) Measurement of luciferase activity in transfected OASF, RASF, and Huh-7.5 cells with the functional and NS5B polymerase-defective (ΔGDD) HCV subgenomic replicon constructs as well as untransfected cells at 4 and 72 h post transfection. (**b**) Immunofluorescent staining for the non-structural viral protein NS5B in the three cell lines at 72 h post transfection with the functional HCV subgenomic replicon constructs as well as untransfected cells. The number of biological replicates was 3 and the number of experimental replicates was 2 (n = 6). (−), untransfected cells, (+) ΔGDD, cells transfected with the mutated HCV-subgenomic replicon unable to efficient replication; (+) subgenome, cells transfected with the functional HCV-subgenomic replicon.

To evaluate whether OASF and RASF cells are permissive for HCV RNA replication we first established a transfection protocol that reliably delivers viral RNA into the cytoplasm of these primary cells. Rather than using our standard approach based on electroporation of cell lines we used lipofection to deliver viral RNA into these cells, and then validated delivery of RNA by using a beta galactosidase-expressing Semliki forest virus replicon. Gal-Semliki-transfected OASF and RASF cells stained positive for beta galactosidase expression 48 h after transfection (data not shown), indicating that our transfection protocol does deliver viral RNA into these cells. The high firefly luciferase activity reading at 4 h post transfection of the three cell types with either the reporter subgenomic replicon or the replicon with inactive polymerase (Luc-NS3-5B∆GDD) further confirms that these cells are transfectable and translate viral proteins (Fig. 3a). At 72 h post transfection, the measured higher firefly luciferase activity in Huh-7.5 cells transfected with the reporter subgenomic replicon compared to OASF and RASF transfected with the same construct and compared to the transfected three cell types with the truncated replicon construct clearly reflects that OASF and RASF do not support HCV replication (Fig. 3a). This finding was further confirmed by immunoflorescence detection of the HCV-NS5A protein at 72 h post transfection (Fig. 3b), where strong fluorescence was noted only in Huh-7.5 cells and not in OASF or RASF.

### Culture supernatants of RASF and OASF do not block HCV replication in Huh-7.5 cells

**Figure 4:**
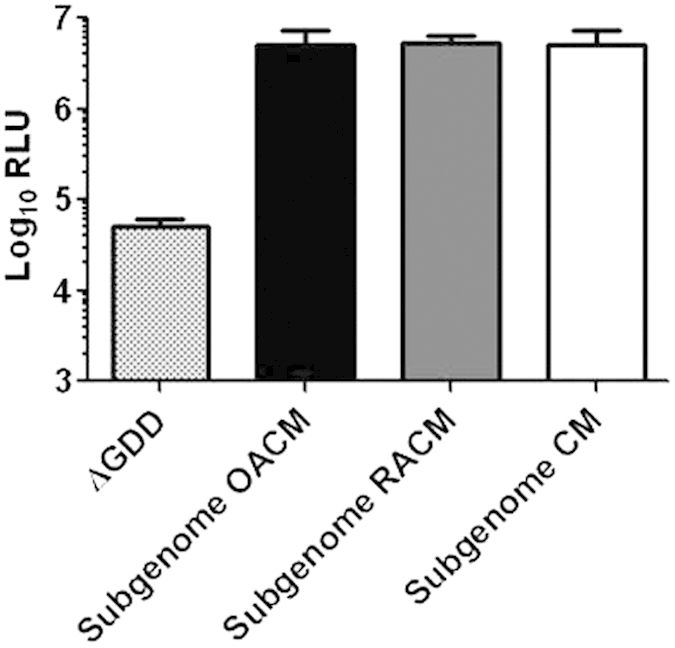
Effect of OASF or RASF culture supernatants on HCV replication in Huh-7.5 cells. Regular culture medium or supernatants from OASF and RASF cultures were incubated with Huh-7.5 cells transfected with the Luc-reporter HCV-subgenomic replicon (subgenome) or the truncated construct (ΔGDD). Luciferase activity was measured 72 h post transfection. The number of biological replicates was 3 and the number of experimental replicates was 2 (n = 6). Subgenome CM: cells transfected with the functional subgenomic replicon with medium replaced 4 h post transfection with regular culture medium. Subgenome OACM and subgenome RACM: cells transfected with the functional subgenomic replicon with medium replaced 4 h post transfection with supernatants from 5-day OASF or RASF cultures.

When Huh-7.5 cells transfected with the HCV luciferase reporter subgenomic replicon were co-incubated with supernatants from either RASF culture medium (RACM) or OASF (OACM) cultures, luciferase activity did not differ from that measured in transfected Huh-7.5 cells co-incubated with control medium (Fig. 4). These results suggest that OASF and RASF do not release any factors that interfere with HCV replication.

### OASF and RASF express lower miR-122 levels than Huh-7.5 cells

**Figure 5:**
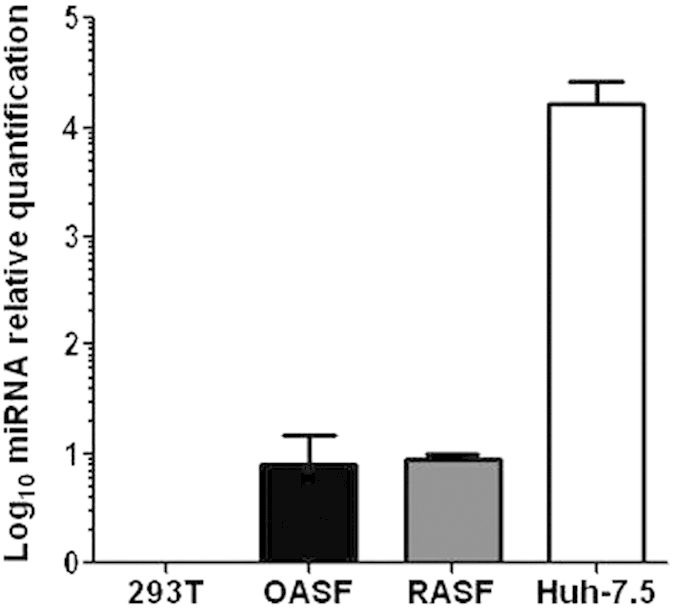
Quantification of miR-122 levels in OASF, RASF and Huh-7.5 cells by quantitative RT-PCR. The number of biological replicates was 3 and the number of experimental replicates was 2 (total n = 6).

HCV replication is strongly facilitated by the presence of miR-122^25^. Quantification of miR-122 in RNA from OASF, RASF and Huh-7.5 cells showed that OASF and RASF contain much lower levels of this miRNA species than Huh-7.5 cells (Fig. 5), suggesting that low levels of miR-122 may be one of the reasons why OASF and RASF do not support HCV replication^26^. Human Embryonic Kidney (293T) cells served as a negative control.

## Discussion

In this study, we examined the permissiveness of human primary OASF and RASF cells for HCV cell entry and RNA replication using a combination of mRNA and protein expression analyses as well as HCV infection and replication assays. Our data provide strong evidence that OASFs and RASFs–when cultured *ex vivo*–restrict viral propagation at multiple steps of the viral life cycle.

First, we observed only very low levels of CLDN1 mRNA in OASF and RASF. In contrast, mRNA levels of OCLN and SR-BI were detectable up to 10^6^ copies per μg total RNA in both cell-types. However, compared to the positive control (Huh-7.5) this was 2-3 log_10_ lower. Additionally, we were unable to detect expression of these critical HCV cell entry factors at the protein level by Western blot analysis. Notably, lack of any one of these cell entry factors has been shown to render cells refractory to HCV infection^24^,^27^,^28^,^29^,^30^. In accordance with this expression analysis, HCV pseudo-particles were unable to infect OASF or RASF^27^. Since pseudo-particles bearing the VSV-G protein were able to infect these cells we can rule out that these cells are generally refractory to retroviral transduction. Second, using a cell transfection-based HCV replication assay involving luciferase-expressing HCV replicons, we did not obtain evidence of productive HCV RNA replication. We confirmed that viral RNA can be delivered to primary OASF and RASF cells by using a Semliki forest virus reporter replicon. Moreover, also 4 h after transfection with the HCV replicon, we measured luciferase activity approx. 10-fold above the background of the assay determined in mock transfected cells. Therefore, OASF and RASF cells can be transfected with HCV RNA and this input RNA is also translated into proteins. However, unlike in the highly HCV permissive Huh-7.5 cells, luciferase activity did not increase between 4 h and 72 h after transfection. In fact, there was no difference between luciferase activity upon transfection of the replication incompetent polymerase defective replicon variant and the replication competent viral RNA at 4 and 72 h after transfection. Therefore, primary OASF and RASF cells are refractory or at least poorly permissive for HCV RNA replication. Interestingly, we found very low expression levels of miR-122 in OASF and RASF. As this liver-specific microRNA is critical for efficient HCV replication^31^, this could be an important molecular determinant for the refractory HCV replication in these cells.

Taken together, the results suggest that human OASF and RASF cells cultured *ex vivo* do not sustain entry or replication of HCV due to lack or poor expression of critical cell entry factors and miR-122.

The absence of any blocking effect of growth media of either OASF or RASF on viral replication in transfected Huh-7.5 cells with the HCV subgenomic replicon also excludes the possibility that synovial fibroblasts release an inhibitor of viral replication.

Our results indicate that synovial fibroblasts do not support HCV infection. Thus, the inflammation of HAA unlikely results from viral infection of this cell type. Which other synovial cell type might support HCV infection? It has been reported that human macrophages are susceptible to HCV infection^32^. As macrophages constitute a significant fraction of synovial cell populations and have been described in increased numbers in HAA^11^, it will be important to check for the presence of HCV RNA and/or proteins in synovial macrophages from patients with HAA (for instance in synovial biopsies), or to isolate synovial macrophages from HAA patients for *ex vivo* studies. Lastly, HAA may develop due to indirect, long distance effects such as the deposition of immune complexes in the synovial membrane of HCV-infected individuals. Further studies, involving synovial tissue and fluid from well characterized patients with HAA, are needed to test these hypotheses.

## Additional Information

**How to cite this article**: Nadeem, A.-E. D. *et al.* Cell culture-derived HCV cannot infect synovial fibroblasts. *Sci. Rep.* **5**, 18043; doi: 10.1038/srep18043 (2015).

## Supplementary Material

Supplementary Information
