# Erratum: Cell culture-derived HCV cannot infect synovial fibroblasts

**DOI:** 10.1038/srep21441

**Published:** 2016-08-26

**Authors:** Dina N. Abd-Elshafy, Thomas Pietschmann, Ulf Müller-Ladner, Elena Neumann, Anggakusuma, Mahmoud M. Bahgat, Frank Pessler, Patrick Behrendt

Scientific Reports 5: Article number: 1804310.1038/srep18043; published online: 12 08 2015; updated: 08 26 2016

The original version of this Article contained errors in the spelling of the authors Dina N. Abd-Elshafy, Thomas Pietschmann, Ulf Müller-Ladner, Elena Neumann, Mahmoud M. Bahgat, Frank Pessler and Patrick Behrendt which were incorrectly given as Abd-Elshafy D. Nadeem, Pietschmann Thomas, Müller-Ladner Ulf, Neumann Elena, Bahgat M. Mohamed, Pessler Frank & Behrendt Patrick respectively.

The ‘How to cite this article’ section quoted an incorrect abbreviation for Dina N. Abd-Elshafy.

In addition, there were errors in the Author Contributions Statement,

“A.D. wrote the initial draft of the manuscript and modified by P.B. Experiments were planned and performed by A.D. and P.B. Initial figure drafts were created mainly by BM (Figures 1–4) and A.K. (Figure 5) and modified by P.B., M.U. and N.E. generated the primary cells and carefully reviewed and edited the manuscript. P.T. and P.F. substantial contributed to the experimental set-up, the manuscript and figure structure. All authors reviewed the manuscript before submission.”

now reads:

“D.A. and M.B. wrote the initial draft of the manuscript and this was modified by P.B. Experiments were planned and performed by D.A. and P.B. Initial figure drafts were created mainly by M.B. (Figures 1–4) and A.K. (Figure 5) and modified by P.B., U.M. and E.N. generated the primary cells and carefully reviewed and edited the manuscript. M.B., T.P. and F.P. substantially contributed to the initial idea, the experimental set-up, the manuscript and figure structure. All authors reviewed the manuscript before submission.”

These errors have now been corrected in the PDF and HTML versions of the Article.

