# Supplementary material for: Cell culture-derived HCV cannot infect synovial fibroblasts
Source: Sci Rep. 2015 Dec 8;5:18043. doi: 10.1038/srep18043 (PMC4672287; doi:10.1038/srep18043)
Supplement: Supplementary Information [file srep18043-s1.pdf]

Cell culture-derived HCV cannot infect synovial fibroblasts

Abd-Elshafy D. Nadeem[1,2], Pietschmann Thomas[3,4], Müller-Ladner Ulf[5], Neumann Elena[5], Anggakusuma[3], Bahgat M. Mohamed[1,6,7], Pessler Frank[1,8], Behrendt Patrick[3,4,9]

[1] TWINCORE, Center for Experimental and Clinical Infection Research, Institute for Experimental Infection Research, Hannover, Germany

[2] Water Pollution Research Department, Environmental Sciences Research Division, National research center, Cairo, Egypt

[3] TWINCORE, Center for Experimental and Clinical Infection Research, Division of Experimental Virology, Hannover, Germany

[4] German Centre for Infection Research, Hannover, Germany

[5] Department of Internal Medicine and Rheumatology, Justus-Liebig University of Giessen, Rheumatology and Clinical Immunology, Kerckhoff Klinik, GmbH, Bad Nauheim, Germany

[6] Therapeutic Chemistry Department, Pharmaceutical Industries Research Division, National research center, Cairo, Egypt

[7] Immunology and Infectious Diseases Laboratory, Therapeutic Chemistry Department, the National Research Centre, Cairo, Egypt

[8] Helmholtz Centre for Infection Research (HZI), Braunschweig, Germany

[9] Department for Gastroenterology, Hepatology and Endocrinology, Medical School Hannover, Hannover, Germany

Keywords:

Hepatitis C, synovial fibroblasts, arthritis

Correspondence:

TWINCORE Center for Experimental and Clinical Infection Research

Division of Experimental Virology

Patrick Behrendt, MD

Feodor-Lynen-Str. 7a

30625 Hannover

eMail: patrick.behrendt@twincore.de

phone: +49511-220027168

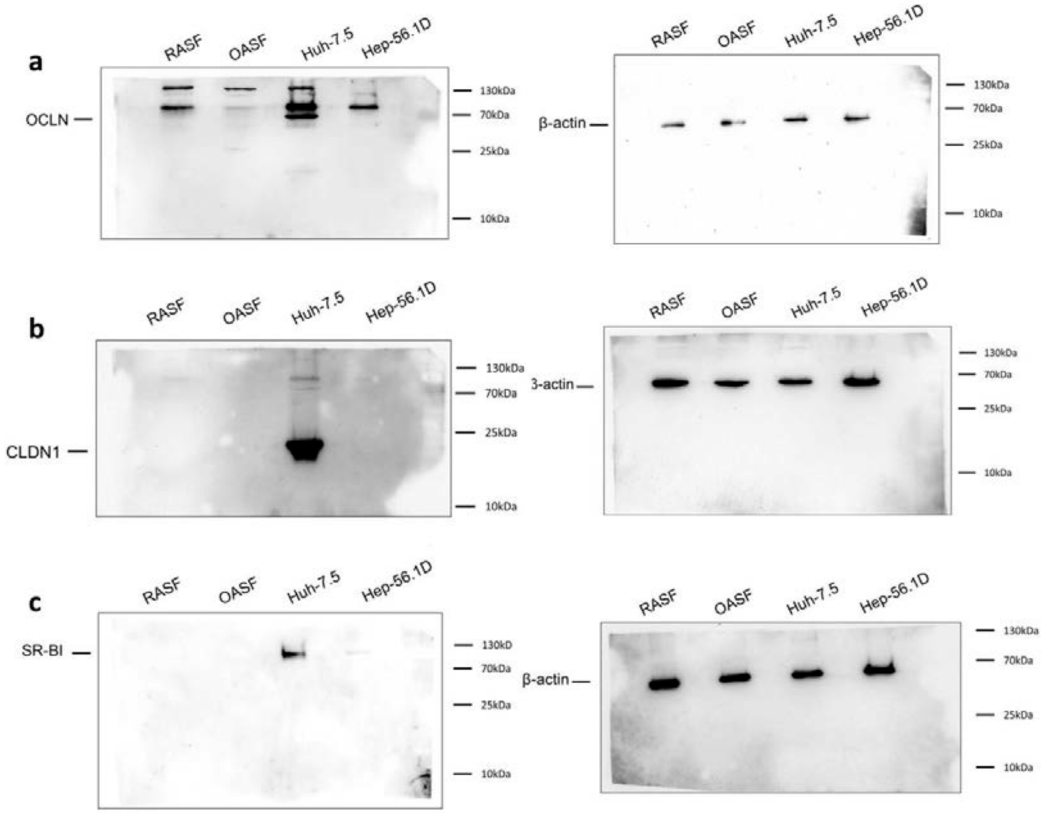

Supplement Figure 1: Full-length immunoblots for detection of OCLN (A), CLDN1 (B) and SR-BI (C) in OASF and RASF. Huh-7.5 cells were used as positive control and Hep-56.1D as negative control. On the right are depicted full-length blots which have been performed in parallel and stained for actin as loading control.
